# Can patient education initiatives in primary care increase patient knowledge of appropriate antibiotic use and decrease expectations for unnecessary antibiotic prescriptions?

**DOI:** 10.1093/fampra/cmae047

**Published:** 2024-09-19

**Authors:** Chloe R Hunter, Katherine Owen

**Affiliations:** Warwick Medical School, University of Warwick, Coventry, United Kingdom; Warwick Medical School, University of Warwick, Coventry, United Kingdom

**Keywords:** antibiotics, antibiotic misuse, antibiotic resistance, patient education, education initiatives, general practice, family practice, systematic review

## Abstract

**Background:**

Healthcare globally is increasingly threatened by antibiotic resistance. Misunderstanding of the appropriate use of antibiotics is common within the general population, therefore patient education could be a useful tool to employ against antibiotic resistance. Patient satisfaction with healthcare is important, and antibiotic awareness is crucial to avoid disappointment when antibiotic stewardship is practiced.

**Aim:**

This review aims to identify whether patient education is an effective tool to improve knowledge and awareness of the appropriate use of antibiotics and whether it has an effect on expectations of or prescription rates of antibiotics.

**Method:**

Embase, Medline, Web of Science, PubMed, and Cochrane Library were searched to identify studies examining the impact of various forms of patient education on awareness of appropriate antibiotic use and antibiotic prescription rates. Reference lists of eligible studies were also screened.

**Results:**

Three hundred and fourteen unique studies were identified, of which 18 were eligible for inclusion. All studies were of good quality. Three studies examined public health campaigns, five examined leaflets, two examined posters, three examined videos, four used mixed interventions and one study examined a presentation. The results were too heterogenous to perform a meta-analysis.

**Conclusion:**

Patient education is an effective tool to increase public knowledge and awareness of the appropriate use of antibiotics, and can reduce the expectation of or prescription rates of antibiotics. The form of patient education matters, as interventions involving active learning and engagement demonstrate significant positive outcomes, whereas passive forms of learning do not appear to have any effect on understanding or prescriptions.

**Key messages**

- Antibiotic resistance is increasing alarmingly due to misuse and overuse.
- Appropriate antibiotic use is poorly understood by patients.
- Patient education is often underutilized compared to clinician education.
- Form of education matters, with different forms having varying success.
- Educating patients is difficult and requires an active, combined approach.
- Appropriate education successfully alters attitudes towards antibiotic use.

## Background

The World Health Organisation has described antibiotic resistance as a global crisis where we are heading towards a “post-antibiotic era” in which common infections and minor injuries will become deadly once again [1]. Antibiotic resistance is hastened by misuse and overuse and requires interventions at all levels of society [2]. Around 80% of UK antibiotic prescriptions in humans are issued in primary care settings, and it is estimated that rates are similar worldwide [2]. Furthermore, a 2014 study revealed that 55% of GPs reported pressure from patients to prescribe antibiotics, with 45% admitting to prescribing unnecessary antibiotics for a viral infection due to patient demand [3]. The same study found that 4% of adults expect to have antibiotics prescribed on every visit to primary care, and 6% expect antibiotics on most occasions [3]. Butler *et al*. concluded patients associate antibiotic prescriptions with treatment, even when unnecessary, and patients who leave without a prescription feel disappointed in their care, which threatens the doctor-patient relationship [4]. Therefore, primary care settings are an appropriate target for initial patient education attempts due to significant patient demand for antibiotic therapy.

Antibiotic misuse is a complex problem requiring a multi-faceted response. Therefore, this systematic review aims to synthesize the available data on the impact of various forms of patient education on patient knowledge and understanding of appropriate antibiotic use to assess whether patient education has a positive impact on reducing unnecessary antibiotic prescriptions.

## Methods

### Search strategy

This study is a systematic review aiming to synthesize and analyse available research to identify whether patient education can have a significant effect on antibiotic awareness and prescriptions and, if so, establishing the most effective methods of achieving this. This study design was chosen to allow for an unbiased synthesis of all currently available evidence to provide a robust answer to the research question. This review is registered, and the review protocol can be accessed via author correspondence.

A database search of Embase, Medline, Web of Science, PubMed, and Cochrane Library was performed using the following MeSH terms: Patient Education, Drug Prescriptions, General Practice, Antibiotic Resistance, and Anti-bacterial Agents. The following free terms were searched with the previous MeSH headings: Patient Information, Practice Patterns, Drug Utilization, Prescri*, Drug Resistance, Microbials, Antibiotics.

### Inclusion and exclusion criteria

Following the database search, a primary screen of the title and abstract of each article was carried out against the inclusion and exclusion criteria. Inclusion and exclusion criteria, outlined in Table 1, were chosen to allow the review to examine patient education initiatives in isolation. It was decided to exclude papers that were published prior to 2000 due to the technological advances that have allowed for multiple methods of education to become more mainstream and readily accessible, such as online resources and interactive web-based resources. It was also decided to limit included papers to those published in English only, as it was found that this restriction allowed for studies from across four continents to be included but avoided issues of translation. Next, a secondary screen was carried out where the remaining full papers were read and compared with the same inclusion and exclusion criteria. The reference lists of the selected studies were screened for additional studies according to the same criteria. All eligible studies were appraised using the Mixed Methods Appraisal Tool [5]. To ensure the validity of the results, the entire search process was repeated by the original reviewer 3 weeks after the initial search. Where there was uncertainty, these studies were discussed with a second reviewer.

**Table 1.** Inclusion and exclusion criteria.

Inclusion criteria	Exclusion criteria
English language Published after 2000 Primary research Examines patient knowledge and attitudes towards antibiotics and/or antibiotic prescribing rates and/or patient expectations of antibiotics. Intervention is a form of patient education Primary care setting	Non-English language Published prior to 2000 Secondary research Non-antibiotic related Secondary or tertiary care settings

### Data extraction

A data extraction table was used to collate the relevant findings from each study, using the headings: Author, Year, Title, Country, Type of Study, Sample Size, Methodology, Intervention, Primary Outcomes, and Secondary Outcomes. The primary and secondary outcomes included the impact of patient education on patient awareness, knowledge, and attitudes towards antibiotics, and the impact of the intervention on antibiotic prescriptions or reported expectations of antibiotics. A meta-analysis of the studies was not performed due to the heterogenous nature of the reported data.

## Results

### Study selection

The database search identified 449 papers, of which 135 were duplicates, leaving 314 papers. The primary screen of titles and abstracts excluded 256 papers, leaving 58 papers to be read in full. Following the second screen, 17 papers remained eligible. An additional 2 papers were identified during a reference list screen. A total of 18 studies met the inclusion criteria for this review: 1 qualitative, 1 mixed methods, and 16 quantitative studies.

### Study characteristics

A table of study characteristics comprising of authors, date, location, study design, intervention, sample size, and outcome measures was created and is shown in Supplementary Appendix 1.

### Quality

To assess the quality of the selected papers and identify potential weaknesses or biases, the Mixed Methods Appraisal Tool [5] was used. The full quality assessment table is shown in Supplementary Appendix 2.

### Study findings

To compare the results, the studies were grouped based on the intervention examined. Six categories were identified: public health campaigns, leaflets, posters, videos, mixed methods, and other. As the methodology, interventions, and results were too heterogenic to accurately compare, a meta-analysis was not performed. Data were extracted on patient knowledge and awareness of antibiotic use and the effect of the intervention on patient use or expectation of antibiotics, or the effect of the intervention on antibiotic prescription rates. The full data extraction table is shown in Supplementary Appendix 3.

## Results

### Public health campaigns

Overall, the three public health campaigns studied [6–8] were largely ineffective in changing public understanding and attitudes towards antibiotics. McNulty *et al*. [6] and Parsons *et al*. [8] examined antibiotic campaigns in England and concluded there was no significant change in public understanding of antibiotic use. Similarly, Curry *et al*. [7] examined a campaign in New Zealand and concluded that there was “no change” in public understanding of the use of antibiotics. Each campaign used similar methods of education, such as radio broadcasts, posters, and leaflets, and each study reported low levels of public familiarity with the campaigns. However, Curry *et al*. [7] achieved responses from 200 people in the pre- and post-intervention surveys. Although the response rate was sufficient for the survey, a sample size of 200 people may not be adequate to assess the impact of a country-wide public health campaign. Parsons *et al*. [8] achieved responses from 442 people in the pre-intervention survey and 815 in the post-intervention survey when analysing a specific borough, which appears a more reliable sample size to adequately assess the effect of a local campaign.

### Leaflets

Five studies examined the effect of an information leaflet [9–13]. Of these, four found an overall positive effect of the information leaflet, and two demonstrated limited results. Min Lee *et al*. [9] reported that whilst patients stated the pamphlet increased their understanding of antibiotics, 20.6% of intervention patients received unnecessary antibiotics for their symptoms compared to 17.7% of control patients. However, the study found when comparing ethnic groups, Indian intervention patients received significantly fewer antibiotics (OR 0.28, 95% CI 0.09–0.93). This study was carried out in Singapore, and participants were from Chinese, Malay, or Indian backgrounds. However, the information leaflet was published in English only, leading the authors to suggest that language barriers and varying English language proficiency may have affected the results, particularly as a positive effect was seen in one ethnic group.

### Posters

Two studies examined the effect of posters [14, 15]. One study found a significant positive effect, whereas the second found the intervention had no effect. Ritchie *et al*. carried out a pre-and post-intervention survey after showing patients a randomly allocated poster, which found viewing posters halved patient expectations to receive antibiotics for a “bad cold” from 27% pre-intervention to 13% post-intervention [14]. Contrastingly, Ashe *et al*. examined the effect of a waiting room poster on antibiotic use and found no significant difference in antibiotic prescribing rates between intervention and control months, concluding the poster was ineffective in reducing paediatric antibiotic prescriptions [15].

### Videos

Three studies examined the impact of videos [16–18]. Of these, one reported significant positive effects on patient expectations of antibiotics, one reported mixed effects and one study reported a modest impact. Lecky *et al*. examined the impact of five 30-second-long video animations on patient awareness of antibiotics and attitude towards their use [16]. This mixed-methods study reported that patients found the animations memorable and informative, with quotes from patients demonstrating understanding of the messages in the animations, and saw significant positive effects on adult patient’s intentions to use antibiotics [16]. However, this intervention had a limited effect on parent’s intentions to consult for their children with similar symptoms. Bauchner *et al*. saw no overall difference between intervention and control groups, however, subgroup analysis revealed an improvement in knowledge in intervention groups in an urban clinic compared to little difference in test scores in the suburban clinic [17]. Contrastingly, Wheeler *et al*. examined the impact of a waiting room video message on parental attitudes towards antibiotic use in children and found that parents who viewed the intervention were significantly less likely to seek antibiotics for viral infections [18]. This video intervention was eight minutes long and featured local doctors and nurses with a segment delivered by an infectious disease expert [18]. This intervention may be seen as more trustworthy by parents than brief, humorous animations, as the intervention was delivered by healthcare professionals known to the participants which could improve the effectiveness of the intervention. Furthermore, the sample size of 771 used by Wheeler *et al*. [18] was greater than the sample size of 56 used by Lecky *et al*. [16], and consisted solely of parents, therefore this study could have greater validity when applied to paediatric patients compared to Lecky *et al*. [16].

### Mixed interventions

Four studies used multiple interventions to assess the impact of patient education on antibiotic awareness and use [19–22]. McNicholas and Hooper used posters, GIFs, and memes displayed in examination rooms [19]. This study found the rate of total and repeat consultation prescriptions for antibiotics decreased significantly after introducing the patient education materials [19]. Gonzales *et al*. provided educational materials for primary care settings, consisting of waiting room and examination room posters and reference cards, and household educational materials consisting of bilingual brochures, reference cards, and a letter explaining the Be S.M.A.R.T campaign [20]. This study found the campaign had a considerable effect on antibiotic prescribing for adults with acute bronchitis, but a negligible effect on prescribing for paediatric pharyngitis, in keeping with the apparent trend of patient education initiatives seemingly having a limited impact on paediatric prescribing [20].

Taylor *et al*. carried out an RTC to evaluate the effectiveness of an educational pamphlet and video on parental attitudes about antibiotic use in children and found a significant difference in parental opinion on the correct use of antibiotics post-intervention [21]. Using the same data, Taylor *et al.* evaluated the effectiveness of the same intervention in reducing antibiotic use in children and found no statistically significant decrease in antibiotic prescription between the intervention and control arms [22]. Therefore, while the educational materials improved parents’ understanding of antibiotic use, the materials did not reduce consultations or prescriptions for unnecessary antibiotics for viral infections in children.

### Other

Perera *et al*. carried out an RCT to assess the impact of a six-slide presentation on patient expectations of antibiotics for upper respiratory tract infections [23]. The study found that viewing one of the intervention presentations halved the reported patient expectation for antibiotics [23]. This intervention also demonstrated a reduction in expectations for antibiotics when the results were restricted to examining the 91 child participants, demonstrating an overall positive effect [23].

## Discussion

The results show that patient education is an effective method of increasing knowledge and reducing antibiotic prescriptions in adults, if an effective method is chosen. However, in paediatric prescribing, the results demonstrate that patient education is less effective. The interventions which demonstrated the most significant effects in both adult and paediatric populations involved active forms of education, such as interactive leaflets and animations, where the participants were engaged with the intervention and often discussed the intervention with either their doctor or the researchers.

Research supports that “active learning” techniques improve student performance when compared to traditional lecture-style teaching [24]. “Active learning” is “any instructional method that engages students in the learning process” as opposed to passively receiving information [25]. Therefore, patients should also be encouraged to take an active role in education interventions. Supporting this, a systematic review of effective strategies for patient education concluded that verbal education alone is the least effective strategy, whereas written materials, videotapes, and multiple strategies were more effective, with 62% of patients who received patient education using multiple strategies having better outcomes than those who received standard care [26]. This, therefore, helps explain the varying results of the studies examined in this review. For example, Ashe *et al*. [15] and Ritchie *et al*. [14] both examined the impact of posters as a form of patient education. However, Ashe *et al* [15]. made no attempt to encourage patients to view the poster, and it is unclear whether patients viewed the poster, affecting the validity of the results. Contrastingly, the methodology used by Ritchie *et al*. [14] involved specifically handing patients different posters and encouraging them to read and discuss the content with the researchers, therefore engaging and involving the patients in the intervention. This is similarly seen in the four successful leaflet interventions [10–13], as each of these interventions involved the patient being directed to an educative resource and taking an active role in the discussion.

Similarly, when examining health beliefs and behavioural change, the Health Belief Model (HBM) [27] and the Transtheoretical Model of Behavioural Change (TMBC) [28] can help identify how to best target patient education to maximize the effectiveness of education interventions. The HBM explains how a person’s action depends on their perception of the benefits and barriers related to the health behaviour [27]. In this case, a person must understand the risks of antibiotic misuse and the benefits of appropriate antibiotic use, alongside recognizing the threat of antibiotic resistance and the side effects of unnecessary antibiotic prescriptions, compared to a potential belief that antibiotics are necessary for viral symptoms. Similarly, the TMBC outlines the stages that a person undergoes when making a change. In order to enact behavioural change, a person must enter the “contemplation” stage, where they identify a problem and start to seek a solution. A study found that 47.8% of respondents incorrectly identified antibiotics as being effective in treating viral infections [29]. Therefore, it is reasonable to suggest that this population would not enter this “contemplation” stage as they do not see antibiotic misuse as a problem that applies to them, as they believe they are seeking antibiotics for a genuine need. This may further explain why the passive interventions outlined in this review had limited impact, as these interventions relied on people not only engaging with the intervention independently, but being able to apply the intervention to their circumstances and recognize it as being applicable to their situation in order to begin a cycle of behavioural change. As previously explained, the three public health campaigns studied [6–8] proved largely ineffective. Despite their broad range of interventions, such as broadcasts, adverts, and posters, these interventions required people to independently engage with and consider the messages. Contrastingly, the more effective interventions, such as the interactive presentation studied by Perera *et al*., specifically linked the patient’s symptoms and the futility of antibiotic treatment, whilst also providing information on suitable alternative treatments [23]. This enables patients to make the connection between the symptoms they are experiencing and the need to change their expectations for antibiotics, allowing them to start the process of behavioural change. This demonstrates the importance of delivering patient education interventions in a manner where the patient is actively involved in the intervention, to allow them to recognize the issue, apply it to their situation, and modify their behaviour.

Interestingly, studies involving paediatric patients proved largely ineffective, with the exceptions of Francis *et al*. [11] and Johnson *et al*. [10]. The design of these studies allowed the participants to receive education on appropriate antibiotic use and alternative remedies during a consultation, and consolidate this with discussion and educational leaflets. In these examples, the participants are actively shown how their symptoms do not need antibiotics through education and discussion. Contrastingly, the interventions designed by Ashe *et al*. [15] and Taylor *et al*. [22] required participants to independently engage with and apply the interventions, demonstrating a passive form of education with limited effectiveness. Furthermore, the studies that demonstrated a positive effect allowed parents to discuss their concerns with a doctor, which may increase trust in the intervention as it is supplemented with support from a trusted healthcare professional. For example, Taylor *et al*. [21] posted their educational intervention to parents, and doctors were blinded as to participation status, removing the ability to use the intervention in a consultation, unlike the methodology of Johnson *et al*. [10]. Interestingly, the results of Taylor *et al*. [22] and Taylor *et al*. [21] demonstrated a positive change in parental attitudes towards antibiotic use, but no change to prescribing or consultation rates, meaning it is unclear whether the posted leaflet intervention was sufficient to allow the parents to associate the information provided with the symptoms experienced by their child. Similarly, Min Lee *et al*. [9] used medical students to hand out leaflets to patients independently of their consultation, whereas the four leaflet interventions with positive effects [10–13] incorporated their leaflets into the consultation. Therefore, these studies further demonstrate the benefit of an active approach to patient education and suggest that more education and resources are needed to be effective in paediatric consults to reassure parents.

While this review grouped studies to allow for effective comparison of the results, it is clear that one common characteristic unites the most effective interventions—the extent to which the patient is involved in the intervention. Studies that proved entirely ineffective, such as Ashe *et al*. [15] from the poster category, McNulty *et al*. [6] from the public health campaign category, and Min Lee *et al*. [9] from the leaflet category, all demonstrated passive methodologies, with each study relying on the patient taking the initiative to independently engage with the resource provided. The studies that saw significant effects, such as Ritchie *et al*. [14] from the poster category, Perera *et al*. [23] from the mixed interventions category, and Johnson *et al*. [10] from the leaflet category, all directed patients to view specific educative initiatives which were relevant to them, engaged them in discussion and allowed patients to take an active role in their learning and be fully involved in the delivery of the intervention. Studies that had more moderate effects, such as Bauchner *et al*. [17] from the video category, did attempt to engage patients in targeted video interventions, but did not also engage the patients in discussion or provide further information to consult at a later date. This demonstrates that whilst these patients were directed to watch a specific, targeted video, they were not offered the opportunity to consolidate this with discussion or further resources, limiting the involvement of the patient in the intervention, and thus limiting the effectiveness. Therefore, the most effective patient education initiatives provide opportunities for active learning and seek to actively involve the patient in the process.

### Comparisons

To the best of our knowledge, this is the first review to synthesize the effectiveness of multiple patient education initiatives on the understanding and use of antibiotics, however, many studies investigating the efficacy of patient education materials on common conditions, management options, and health promotion were identified during the initial literature search. A systematic review on providing patient education materials for non-specific lower back pain found a high degree of variability across outcomes but concluded providing materials is favourable [30]. Similarly, a one-year prospective study found a patient education course improved asthma control and decreased exacerbations in patients with severe uncontrolled asthma [31]. With regards to antibiotics, a systematic review by de Bont *et al*. concluded that patient information leaflets are effective in reducing antibiotic prescriptions, actual antibiotic use, and intentions to reconsult for future similar symptoms [32]. These studies further demonstrate that patient education is an effective and valuable tool, as found in this review, and support the suggestion that patient education is an effective and useful tool to implement in the strategy against antibiotic resistance.

### Practical and theoretical implications

This review demonstrates patient education is a useful tool to improve patient understanding and awareness of antibiotic use, which can be used to further inform antibiotic awareness campaigns and support antibiotic resistance strategies. In practice, educative initiatives have been shown to be useful with minimal impacts on patient satisfaction, indicating these interventions should be introduced into primary care more widely.

### Strengths and limitations

The main strength of this review is the search strategy, as five databases were searched, allowing for a thorough literature review. However, this review is limited as the studies included were conducted in Western populations, particularly the USA. Therefore, the results may not be culturally applicable worldwide. This review is further limited by the heterogeneity of the data, as differences in population samples, primary and secondary outcomes, and interventions make statistical analysis more difficult. Finally, due to limited resources, only one reviewer carried out the search and data extraction. Although this was repeated to ensure the validity of the results, a secondary reviewer would have been beneficial.

### Future work

As this review demonstrates patient education is an effective tool in improving antibiotic awareness and use, it would be useful to carry out further trials directly comparing education interventions to determine the most effective and cost-effective interventions.

## Conclusion

Patient education initiatives are an effective way to improve patient awareness of antibiotic misuse and reduce antibiotic prescriptions, if they are delivered in an effective manner. Not all forms of education are equal, and the most significant results were demonstrated by initiatives that sought to involve and engage the patient in the educative intervention.

## Supplementary material

Supplementary material is available at *Family Practice* online.

cmae047_suppl_Supplementary_Appendix

## Data Availability

All data, including full search terms and eligibility criteria, are available either in the main article or in the supplementary materials submitted with the manuscript.
