# Supplementary material for: Can patient education initiatives in primary care increase patient knowledge of appropriate antibiotic use and decrease expectations for unnecessary antibiotic prescriptions?
Source: Fam Pract. 2024 Sep 19;42(2):cmae047. doi: 10.1093/fampra/cmae047 (PMC11878379; doi:10.1093/fampra/cmae047)
Supplement: cmae047_suppl_Supplementary_Appendix [file cmae047_suppl_supplementary_appendix.zip › Appendix 1 pdf.pdf]

Appendix 1: Study characteristics table including location, study design, intervention, sample size and outcome measures.

Authors & Date	Location	Study Design	Intervention	Sample size	Outcome Measures
Johnson et al. (2023).	USA	Pre-post survey	Modified CDC antimicrobial stewardship teaching leaflet	56	Patient knowledge on appropriate antibiotic use.<br><br>Patient expectations of antibiotic prescription.
McNicholas & Hooper (2022).	USA	Mixed methods	CDC Be Antibiotics Aware posters, GIFs and memes	250 (188 control, 62 intervention)	Antibiotic prescription rate.
Perera et al. (2021).	New Zealand	RCT	6 slide presentation about either:<br>1. The futility of antibiotic treatment for URTIs and information about alternative treatments<br>2. The potential adverse effects of antibiotics and information about suitable alternative treatments for URTIs<br>3. Control presentations promoting healthy lifestyle choices	325 (102 control, 119 futility, 104 adverse effects)	Patient knowledge on appropriate antibiotic use.<br><br>Patient expectations of antibiotic prescription and intention to consult for antibiotics.
Ritchie et al. (2019).	New Zealand		3 posters with the same font, layout and colour scheme:<br>1. Poster focused on the 'futility' of antibiotic treatment for URTIs	299	Patient knowledge on appropriate antibiotic use.<br><br>Patient expectations of antibiotic prescription and intention

			2. Poster focused on the frequency of adverse drug reactions of antibiotics<br>3. Poster focused on the development of antibiotic resistance		to consult for antibiotics.
Lecky et al. (2017).	England	Mixed methods	5 30-second-long animations about prudent antibiotic use displayed on waiting room TVs every 20 minutes	132	Patient knowledge on appropriate antibiotic use.<br><br>Patient expectations of antibiotic prescription and intention to consult for antibiotics.
Min Lee et al. (2017).	Singapore	RCT	Educational pamphlets and verbal counselling on the causes of URTIs and the role of antibiotics in treating URTIs	916 (457 control, 457 intervention)	Patient understanding of antibiotics and viral disease.<br><br>Antibiotic prescription rates for viral disease.
McNulty et al. (2010).	UK	Random location sampling survey	English Antibiotics Campaign 2008	1888 pre-intervention, 1830 post-intervention	Patient knowledge and attitudes towards antibiotic use.<br><br>Patient expectations of antibiotic prescription and intention to consult for antibiotics.
Francis et al. (2009).	England & Wales	Cluster RCT	8-page interactive booklet about childhood respiratory tract infections	528	Patient understanding of viral disease and antibiotics.<br><br>Intention to consult for

					viral disease and antibiotic use for viral disease.
Sahlan et al. (2008).	Germany	Qualitative	Adaptation of a German antibiotic awareness leaflet into Turkish to target a high prescription population	57	Patient understanding of appropriate antibiotic use.<br><br>Patient expectations for antibiotic prescriptions.
Curry et al. (2006).	New Zealand	Comparative study	Wise Use of Antibiotics Campaign	Pre-intervention-200<br>Post-intervention - 200	Patient knowledge of viral diseases and antibiotics,<br><br>Patient expectations of antibiotic prescriptions.
Ashe at al. (2006).	USA	Non-randomised control trial	Educational poster displayed in practice waiting area titled 'a parent's guide to help understand colds and viruses'	720	Antibiotic prescribing rates.
Taylor et al. (2005).	USA	RCT	Educational pamphlet 'your child and antibiotics' and a video featuring a doctor from the study child's practice	499	Consultation rates for viral illness.<br><br>Antibiotic prescriptions for viral illness.
Gonzales et al. (2005).	USA	Non-randomised control trial	Be S.M.A.R.T about antibiotics campaign	7 primary care practices	Paediatric and adult antibiotic prescription rates.
Taylor et al. (2003).	USA	RCT	Educational pamphlet 'your child and antibiotics' and a video featuring a doctor from the study child's practice	499	Patient understanding of appropriate antibiotic use.
Parsons et al. (2004).	England (Barking and Dagenham borough)	Questionnaire survey	Local enhancement of the CATNAP campaign	Pre-intervention 450<br>Post-intervention 827	Patient understanding of appropriate antibiotic use.<br><br>Patient attitudes towards

					antibiotic prescribing.
MacFarlane et al. (2002).	Nottingham, England	RCT	Patient information leaflet about antibiotic use, delayed prescription use and home remedies	259<br><br>(212 given delayed prescription, 106 of these given leaflet, 47 given immediate prescription)	Rate of antibiotic use.
Wheeler et al. (2001).	USA	Prospective cohort study	8-minute video on judicious antibiotic use	771	Patient understanding of appropriate antibiotic use.<br><br>Patient expectations of antibiotic prescriptions.
Bauchner et al. (2001).	USA	RCT	20-minute video on common viral and bacterial childhood infections, differences between bacteria and viruses and correct antibiotic use	193	Patient understanding of appropriate antibiotic use.<br><br>Patient expectations of antibiotic prescriptions.
