# Supplementary material for: Can patient education initiatives in primary care increase patient knowledge of appropriate antibiotic use and decrease expectations for unnecessary antibiotic prescriptions?
Source: Fam Pract. 2024 Sep 19;42(2):cmae047. doi: 10.1093/fampra/cmae047 (PMC11878379; doi:10.1093/fampra/cmae047)
Supplement: cmae047_suppl_Supplementary_Appendix [file cmae047_suppl_supplementary_appendix.zip › Appendix 2 pdf.pdf]

Appendix 2: Study quality assessment using the Mixed Methods Appraisal Tool (Hong et al., 2018).

	Methodological Quality Criteria	Responses			
		Y	N	CT*	Comments
Johnson et al., 2023	Is the sampling strategy relevant to address the research question?	X			
	Is the sample representative of the target population?	X			
	Are the measurements appropriate?	X			
	Is the risk of nonresponse bias low?	X			A response rate of 96.5% was achieved.
	Is the statistical analysis appropriate to answer the research question?	X			
McNicholas & Hooper, 2022	Are the participants representative of the target population?	X			
	Are measurements appropriate regarding both the outcome and intervention (or exposure)?	X			
	Are there complete outcome data?	X			
	Are confounders accounted for in the design and analysis?		X		The outcome measured related to the prescription rates of healthcare providers, whereas the intervention focused on patient education. It is explained in the discussion that it cannot be determined if the reduction in prescribing rates is due to patient education for certain, or whether the education tools aided prescribers in not prescribing antibiotics. However, this is not accounted for in the design or results of the study.
	During the study period, is the intervention administered (or exposure occurred) as intended?	X			
	Is randomisation adequately performed?			X	Study describes that participants were randomised to view 1 of 3 presentations, and describes the allocation as 1:1:1 but there is no

Perera et al., 2021					detail provided on how participants were randomised.
	Are the groups comparable at baseline?	X			Comparable at baseline, but the groups may not have been fully representative of the target population overall as the participants were recruited from 2 practices in wealthy, urban areas serving populations with good English proficiency.
	Are there complete outcome data?	X			Full data obtained for 90% of the cohort.
	Are outcome assessors blinded to the intervention provided?	X			
	Did the participants adhere to the assigned intervention?	X			
Ritchie et al., 2019	Are the participants representative of the target population?	X			No specific inclusion or exclusion criteria were used; however, all participants were recruited from the same adult inpatient general medical, surgical and orthopaedic wards at Auckland City Hospital. All patients present at the time of the study personnel visit were invited to participant except those deemed too unwell by ward staff. Demographic information provided by the researchers appears broadly representative of the New Zealand population.
	Are measurements appropriate regarding both the outcome and intervention (or exposure)?	X			
	Are there complete outcome data?	X			300 participants were invited to participate and 1 was excluded due to being discharged before completing the follow up survey. 299 participants completed the study in full.
	Are confounders accounted for in the design and analysis?	X			
	During the study period, is the intervention administered (or exposure occurred) as intended?	X			
	Is there an adequate rationale for using a mixed methods design to address the research question?	X			

Lecky et al., 2017	Are the different components of the study effectively integrated to answer the research question?	X			
	Are the outputs of the integration of qualitative and quantitative components adequately interpreted?	X			
	Are divergences and inconsistencies between quantitative and qualitative results adequately addressed?	X			
	Do the different components of the study adhere to the quality criteria of each tradition of the methods used?	X			
Min Lee et al.,2017	Is randomisation adequately performed?	X			Participants were randomised using sequential envelopes containing completer generated assignments based on simple block randomisation. Randomising at the patient level was chosen to avoid the confounding effect of variations in GP antibiotic prescription practices.
	Are the groups comparable at baseline?	X			
	Are there complete outcome data?	X			1 participant from each arm did not complete the study so 457/458 participants completed each arm of the study.
	Are outcome assessors blinded to the intervention provided?	X			Researchers were allocated in pairs – the first researcher carried out the pre-consultation questionnaire and handed out the randomised envelope containing the intervention or control leaflet. The second researcher was not involved in the pre-consultation questionnaire or randomisation carried out the post-consultation questionnaire.
	Did the participants adhere to the assigned intervention?	X			
McNulty et al., 2010	Are the participants representative of the target population?	X			Random location sampling and non-random quota sampling was used.
	Are measurements appropriate regarding both the outcome and intervention (or exposure)?	X			
	Are there complete outcome data?	X			

	Are confounders accounted for in the design and analysis?	X			
	During the study period, is the intervention administered (or exposure occurred) as intended?	X			English antibiotic awareness campaigns in February and November 2008.
Francis et al., 2009	Is randomisation adequately performed?	X			Randomisation was performed by a statistician using block randomisation with random block sizes and stratification by practice list size, antibiotic prescribing rate for 2005 and country.
	Are the groups comparable at baseline?	X			
	Are there complete outcome data?	X			Follow up rate of 94.6%.
	Are outcome assessors blinded to the intervention provided?	X			Assessors were blinded to the intervention, but the interviewers reported becoming aware of which group participants had been allocated to in 34 of 509 interviews (6.7%).
	Did the participants adhere to the assigned intervention?	X			
Sahlan et al., 2008	Is the qualitative approach appropriate to answer the research question?	X			
	Are the qualitative data collection methods adequate to address the research question?	X			Individual interviews were carried out in Turkish (study was carried out in Germany but involved Turkish patients). Interviews used a semi-standardised script using narrative stimuli and lasted between 4 and 12 minutes.
	Are the findings adequately derived from the data?	X			
	Is the interpretation of results sufficiently substantiated by data?	X			Quotes provided justify the themes.
	Is there coherence between qualitative data sources, collection, analysis and interpretation?	X			
	Are the participants representative of the target population?	X			Demographic information provided demonstrated that the randomly chosen sample population was roughly representative of the New Zealand population.

Curry et al., 2006	Are measurements appropriate regarding both the outcome and intervention (or exposure)?	X			
	Are there complete outcome data?	X			Response rate for 1998 survey was 72% and for the 2003 survey was 55% - this is acceptable for the type of study and the researchers saw no reason to indicate that non-responses introduced any bias in the results.
	Are confounders accounted for in the design and analysis?	X			
	During the study period, is the intervention administered (or exposure occurred) as intended?	X			Wise Use of Antibiotics campaign.
Ashe et al., 2006	Are the participants representative of the target population?	X			
	Are measurements appropriate regarding both the outcome and intervention (or exposure)?	X			
	Are there complete outcome data?	X			
	Are confounders accounted for in the design and analysis?	X			
	During the study period, is the intervention administered (or exposure occurred) as intended?	X			The study measured whether a waiting room poster was effective in antibiotic education. In the discussion, the authors noted how they were unable to tell whether patients noticed the poster or understood the information, however the study was designed to assess the efficacy of a poster and this intervention was displayed as intended.
	Is randomisation adequately performed?	X			Randomisation was based on a computer-generated list of study numbers that were consecutively assigned to enrolled patients, and randomisation was also stratified by practice and in blocks of 10.
	Are the groups comparable at baseline?	X			Parents of children in the control group had higher educational levels than those in the intervention group – unadjusted and adjusted analyses were carried out to counteract this and no significant changes were seen based on parental education level so only unadjusted data is presented in the report.

Taylor et al., 2005	Are there complete outcome data?	X			94.6% of study patients completed the whole 12-month observation period.
	Are outcome assessors blinded to the intervention provided?			X	The study coordinator assessed the details of consultations for each study child however it is not made clear whether the study coordinator knew whether each child was in the control or intervention group when reviewing their records. Paediatricians were not informed of the participation of or randomisation status of the study patients.
	Did the participants adhere to the assigned intervention?	X			
Gonzales et al., 2005	Are the participants representative of the target population?	X			
	Are measurements appropriate regarding both the outcome and intervention (or exposure)?	X			
	Are there complete outcome data?	X			
	Are confounders accounted for in the design and analysis?	X			
	During the study period, is the intervention administered (or exposure occurred) as intended?	X			Bilingual (Spanish and English) education materials were posted to households, and further educational materials were displayed in waiting and examination rooms.
Taylor et al., 2003	Is randomisation adequately performed?			X	Study states that patients were randomised but there is no detail as to how this randomisation was carried out.
	Are the groups comparable at baseline?	X			
	Are there complete outcome data?		X		72% of participants returned the follow up questionnaire. The educational level of parents who completed the follow up questionnaire was significantly higher than that of non-responders.
	Are outcome assessors blinded to the intervention provided?	X			Paediatricians were not told whether their patient was enrolled in the study or the randomisation of any patient
	Did the participants adhere to the assigned intervention?	X			

Parsons et al., 2004	Are the participants representative of the target population?	X			Questionnaires were sent to random samples of the target population in Barking and Dagenham. Demographic information provided indicates that the samples were roughly representative of the borough population.
	Are measurements appropriate regarding both the outcome and intervention (or exposure)?	X			
	Are there complete outcome data?		X		Response rates for the surveys were 46% (initial) and 43% (follow up) – response rates were low, but are similar for both surveys.
	Are confounders accounted for in the design and analysis?	X			
	During the study period, is the intervention administered (or exposure occurred) as intended?	X			
Macfarlane et al., 2002	Is randomisation adequately performed?	X			
	Are the groups comparable at baseline?	X			
	Are there complete outcome data?	X			
	Are outcome assessors blinded to the intervention provided?	X			
	Did the participants adhere to the assigned intervention?	X			
	Are the participants representative of the target population?	X			
	Are measurements appropriate regarding both the outcome and intervention (or exposure)?	X			
	Are there complete outcome data?	X			
	Are confounders accounted for in the design and analysis?	X			In the discussion, the researchers describe the potential confounding factor of their chart review on prescribing practices, and decided to add further chart reviews from periods before the study took place to assess the rate of inappropriate antibiotic prescribing to gain a clearer view

Wheeler et al., 2001					of the practice's prescribing habits pre-intervention.
	During the study period, is the intervention administered (or exposure occurred) as intended?		X		Researchers found through periodic contact with the clinics that during the 9 months that the study ran, clinics became increasingly noncompliant with showing the video intervention and that in the later months of the study researchers increasingly saw their video intervention had been turned off or replaced by clinic staff or patients.
Bauchner et al., 2001	Is randomisation adequately performed?			X	Study states that participants were randomised but there is no detail as to how the randomisation occurred.
	Are the groups comparable at baseline?	X			When comparing the characteristics of the study participants overall, the groups are similar. However, there were differences between the 2 main enrolment sites – children in the urban clinic were more likely to be black (77% vs 1%) and were more likely to be receiving Medicaid (86% vs 3%) than the children from the suburban practice. Parents in the urban clinic were less likely to have attained college or postgraduate education (45% vs 90%) than parents from the suburban practice.
	Are there complete outcome data?	X			94% completed the post-test questionnaire.
	Are outcome assessors blinded to the intervention provided?			X	The study does not mention whether the interviewers were blinded.
	Did the participants adhere to the assigned intervention?	X			42% of parents stated they had watched the video once, and 39% reported watching it 2 to 7 times.
