# Supplementary material for: Can patient education initiatives in primary care increase patient knowledge of appropriate antibiotic use and decrease expectations for unnecessary antibiotic prescriptions?
Source: Fam Pract. 2024 Sep 19;42(2):cmae047. doi: 10.1093/fampra/cmae047 (PMC11878379; doi:10.1093/fampra/cmae047)
Supplement: cmae047_suppl_Supplementary_Appendix [file cmae047_suppl_supplementary_appendix.zip › Appendix 3 pdf.pdf]

Appendix 3: Data Extraction Table

Study	Effect on patient knowledge and awareness of antibiotic use	Effect on patient use or expectation of antibiotics or antibiotic prescription rate	Summary of results	Category Assigned
Johnson et al., 2023	Post-intervention 0.46-point difference on the 5-point Likert scale used to assess whether participants understood the difference between viruses and bacteria.<br><br>Post-intervention 0.54-point difference in response to the statement 'inappropriate antibiotic use can lead to resistance'.<br><br>Respondents demonstrated a total mean pre-intervention knowledge score of 3.57 compared with a total mean post-intervention knowledge score of 4.52.	Mean pre-intervention score of 3.98 and mean post-interventions score of 4.41 in response to the question 'there are other treatments for low grade fever other than antibiotics'.<br><br>For the statement 'I understand when an antibiotic is needed', the pre-intervention mean was 3.95 and the post-intervention mean was 4.52.	The antimicrobial stewardship teaching leaflet improved knowledge of appropriate antimicrobial use for paediatric respiratory illness.	Leaflet
McNicholas & Hooper, 2022	Not assessed as an outcome measure in this study.	Post-intervention, there was a statistically significant decrease in total antibiotic prescription rate of 12.6% compared to the pre-intervention period.<br><br>Pre-intervention, no antibiotics were prescribed to 72.9% of patients compared with 85.5% of patients post-intervention.<br><br>The repeat consultation prescription rate decreased from 12.7% of visits pre-intervention to 0.6% of visits post-intervention.	The authors concluded that displaying education posters, GIFs and memes alongside providing written information and communication skills were effective in reducing unnecessary antibiotic prescribing.	Mixed Interventions
Perera et al., 2021	Post-intervention reduction in mean Likert	Pre-intervention mean Likert scores for	The authors conclude that 'this	Other

	score for agreement with the statement 'I think antibiotics are a helpful treatment for cold/flu':<br>- Control: -0.1 (95% CI -0.2-0.1)<br>- 'futility' intervention: 0.9 (95% CI 0.6-1.2)<br>- 'Adverse effects' intervention: 0.7 (95% CI 0.4-1.1)	intervention and control groups agreement with the statement 'I wish to receive antibiotics for my/my child's cold/flu' was 3.0. Post-intervention, the reduction in control mean was 0.1 (95% CI 0.0-0.3), and reduction in 'futility' intervention mean was 1.1 (95% CI 0.8-1.3) and in 'adverse effects' intervention was 0.7 (95% CI 0.4-0.9).<br><br>Participants in both intervention groups were less likely to continue to expect antibiotics for cold/flu symptoms:<br>- Futility intervention: unadjusted OR 0.45 (95% CI 0.27-0.75)<br>- Adverse effects intervention: unadjusted OR 0.53 (95% CI 0.32-0.86).	study shows that providing brief information to patients about either the futility of antibiotic treatment or the potential adverse effects of antibiotics when they present to their family practitioner with an URTI halves the patient's expectations for antibiotics'.	
Ritchie et al., 2019	Pre-intervention, 40% of respondents agreed that colds were caused by bacteria. Post-intervention, 15% agreed.<br><br>Pre-intervention, 44% agreed that antibiotics are effective treatments for colds. Post-intervention, 8% agreed.<br><br>Pre-intervention, 64% agreed that antibiotic resistance is an important health problem in New Zealand. Post-intervention, 81% agreed.	Pre-intervention, 27% of respondents agreed that they would expect antibiotics for a 'bad cold'. Post-intervention, 13% of respondents agreed with the statement.<br><br>Pre-intervention, 18% agreed that 'I take antibiotics even if there's only a small chance of benefit'. Post-intervention, 8% agreed.	Viewing one of three educational posters reduced participant's expectations to receive antibiotics for a hypothetical cold.	Poster

	Pre-intervention, 33% agreed that New Zealand uses high amounts of antibiotics. Post-intervention, 90% agreed.			
Lecky et al., 2017	Qualitative quotes demonstrate patient engagement with the animations and understanding of the key messages:<br>- ‘it’s not your doctor’s fault he is not prescribing you something you don’t need’.<br>- ‘Leaves you in no doubt that’s what you want (to do)’ – in reference to an animation describing alternatives to antibiotics (‘try rest, plenty of fluids and over the counter remedies such as paracetamol’).	Pre-intervention, 75% of respondents stated that they had asked for antibiotics for previous cold, cough, sore throat or flu symptoms in the last 6 months. Post-intervention, 59.8% reported that they would be less likely to consult a GP for these symptoms, and 63% would be less likely to ask for antibiotics for these symptoms.<br><br>29.5% of respondents stated that they would be less likely to ask for antibiotics for their child (under 5 years) with cough, flu, cold or sore throat symptoms.	Authors concluded that this study demonstrated a simple and relatively inexpensive intervention was successful in positively influencing a patient’s immediate intention to use antibiotics. However, the intervention had little effect on parent’s intention to consult for antibiotics for their children with similar symptoms.	Video
Min Lee et al., 2017	Patients in the intervention group reported that they had an improved understanding of upper respiratory tract infection causes based on their response on a 4-point Likert scale.	20.6% of the intervention group and 17.7% of the control group received antibiotics for upper respiratory tract symptoms.<br><br>In the Indian subethnic group, the intervention group received fewer antibiotic prescriptions – odds ratio 0.28 (95% CI 0.09 – 0.93).	The educational intervention did not reduce antibiotic prescription rates except in patients of Indian ethnicity, where the intervention had a demonstrable association with an increased awareness that antibiotics are not indicated for URTIs.	Leaflet
McNulty et al., 2010	No significant change in responses to 9 out of 10 attitude statements, including ‘antibiotics	28% reported asking their GP for an antibiotic pre-intervention and 29%	Authors concluded that they ‘could not detect any positive	Public Health Campaign

	work on most coughs and colds' (40% versus 37% post-campaign).<br><br>53% of respondents agreed with the statement 'antibiotics can kill viruses' pre-campaign, and 52% agreed post-campaign (P=0.7).	reported asking for antibiotics post-intervention.<br><br>Post-campaign respondents were significantly more likely to disagree with the statement 'resistance to antibiotics is a problem in British hospitals' (30% pre-intervention versus 37% post-intervention).<br><br>Pre-intervention, 8.3% of respondents stated they had taken antibiotics without being told to do so, and 7.8% of respondents post-intervention had done so.	effect of the campaigns.	
Francis et al., 2009	55.3% of the intervention group expressed an intention to consult again if their child had a similar illness (respiratory tract infection) in the future compared with 76.4% of the control group.<br><br>Re-consultation for the same illness episode occurred in 12.9% of the intervention group and 16.2% of the control group.<br><br>The proportion of parents who felt 'very reassured' after their consultation was 72% for the intervention group and 75.3% for the control group, and 85.4% of intervention group parents reported the information they received was 'very useful' or 'useful'.	19.5% of the intervention group had an antibiotic prescribed at the index consultation, compared with 40.8% of the control group.<br><br>22.4% of the intervention group had taken antibiotics within the first two weeks of the initial consultation (including antibiotics prescribed after the initial consultation) compared with 43% of the control group.	The intervention resulted in a reduction in antibiotic prescriptions and a reduced intention to consult in the future without reducing satisfaction with care, however there was only a statistically non-significant reduction in re-consultation rates for the same illness episode following the intervention.	Leaflet
Sahlan et al., 2008	Qualitative quotes relating to patient	Qualitative quotes relating to patient	The authors concluded that the	Leaflet

	understanding of antibiotics:<br>- Patient 16 ‘I thought that antibiotics would cure a cough quicker, but after reading this leaflet I learn that this is not true’.<br>- Patient 28 ‘I think we use antibiotics to no effect (…) and bacteria have become stronger and stronger’.<br>- Patient 28 ‘I didn’t know that antibiotics don’t treat a cough. I had bronchitis in the past and always took antibiotics (…) I thought that I would recover with the help of antibiotics, but after reading this I am confused. I also learned that the cause of the problem were viruses’.<br>- Patient 40 ‘coughing is not a serious illness and coughing throws out microbes’.<br>- Patient 14 ‘I learned that coughing can be helpful and that it should only be treated if one suffers in the extreme’.<br>- Patient 17 ‘In here it says that a cough is helpful, but it is not. It is	expectations of antibiotics:<br>- Patient 45 ‘to push the doctor to prescribe what they want is not good (…) but some doctors prescribe antibiotics just because they think that patients expect it’.<br>- Patient 44 ‘I learned not to pressurise the doctor for antibiotics every time, antibiotics are not always useful’.	leaflet successfully disseminated relevant and unfamiliar information to the target audience.	

	harmful. It should be treated as soon as possible.'<br>- Patient 20 'here it says that coughing may be useful and does not need to be treated (...) but I cannot imagine how a cough can be helpful'.			
Curry et al., 2006	41% of pre-campaign respondents and 38% of post-campaign respondents understood antibiotic efficacy in regard to bacterial and viral infections (P=0.6).	Pre-campaign, 65% of respondents expected to get antibiotics when consulting a doctor, and post-campaign 63% expected antibiotics (P=0.78).	Authors concluded that the study found 'no change' in the percentage of the public with a 'sound understanding that antibiotics are not effective in the treatment of viral infections'.	Public Health Campaign
Ashe et al., 2006	Not assessed as an outcome measure in this study.	During the intervention month (December 2001), 48.3% of consultations for respiratory illnesses resulted in an antibiotic prescription. During the 3 control months, 45% (November 2000), 52.2% (December 2000) and 35.6% (November 2001) of consultations for respiratory illnesses resulted in an antibiotic prescription.	The waiting room poster used in this study aimed at education parents about appropriate antibiotic use was ineffective in reducing paediatric antibiotic prescriptions.	Poster
Taylor et al., 2005	The mean number of visits due to upper respiratory tract symptoms the intervention group was 2.8 ± 3.0, and 2.8 ± 2.8 for the control group.	The mean number of visits per patient where antibiotics were prescribed for otitis media and/or sinusitis: control = 2.1 ± 2.5, intervention = 1.9 ± 2.3.<br><br>The mean total number of antibiotic prescriptions per	The authors concluded that an educational intervention aimed at parents did not result in a decrease in paediatric antibiotic prescriptions, and that the use of antibiotics in	Mixed interventions

		patient was 2.5 ± 2.9 in the control group and 2.2 ± 2.6 in the intervention group.	children with upper respiratory tract symptoms was common.	
Gonzales et al., 2005	Not assessed as an outcome measure in this study.	Paediatric antibiotic prescriptions decreased from 34% to 30% for children at the intervention practices compared to a rate of 37% at local control practices and 39% at distant control practices.<br><br>Adult antibiotic prescription rates for acute bronchitis decreased from 60% to 36% at the intervention practices compared to 45% at local control practices and 44% at distant control practices.	The intervention had a considerable effect on prescribing for acute bronchitis in adults, but a negligible effect on prescribing for paediatric pharyngitis.	Mixed interventions
Parsons et al., 2004	-2% change in the number of respondents believing that antibiotics 'fight all infections including viruses' (95% CI limit -6.7%, 51% vs 49%).<br><br>-4% change in agreement with the statement 'antibiotics will help a cold to get better more quickly' (95% CI limit -8.1%).<br><br>49% of respondents pre- and post-campaign believed antibiotics would help sore throats (excluding tonsillitis) get better more quickly.	90% of respondents believed children should be prescribed antibiotics for an ear infection pre-campaign, and 91% post-campaign.<br><br>33% of respondents agreed a child's parents are the 'best people to decide whether or not they need antibiotics' pre-campaign, and 27% agreed post-campaign.<br><br>27% of respondents pre- and post-campaign agreed they should be able to ask for antibiotics by phone.<br><br>Antibiotic dispensing rate fell nationally by 27% and by 38% in Barking and Dagenham from 1995/6 to 1999/2000,	Authors concluded that there is a 'considerable misunderstanding' over the use of antibiotics for common viral infections, and that the 'CATNAP campaign was not effective in reducing antibacterial dispensing'.	Public Health Campaign

		but the CATNAP campaign does not appear to have affected the rate of decline.		
Taylor et al., 2003	Average 6 point-Likert scale score for the following statements:<br>- Too many children are treated with antibiotics when not necessary (control 4.86, intervention 5.18).<br>- Parents should not try to persuade a doctor to prescribe antibiotics (control 4.99, intervention 5.26).<br>- Giving an antibiotic to a child with cold symptoms can prevent an infection from occurring (control 2.12, intervention 1.86).<br>- Overuse of antibiotics can make bacteria more resistant to antibiotics (control 5.52, intervention 5.78).<br>- It is worth trying an antibiotic when my child has cold symptoms for 5 days (control 2.34, intervention 1.93).<br>- Treatment with antibiotics is necessary when	Not assessed as an outcome measure in this study.	Authors concluded that a simple educational intervention can significantly alter parental attitudes regarding the use of antibiotics in children.	Mixed interventions

	a child's nasal discharge turns from yellow to green in colour (control 3.47, intervention 2.61).<br>- Antibiotics help a child's cold symptoms clear up more quickly (control 2.01, intervention 1.64).<br>- Antibiotics are helpful in treating colds (control 1.87, intervention 1.52).			
Macfarlane et al., 2002	Not assessed as an outcome measure in this study.	47% of the intervention group took antibiotics compared to 62% of the control group.<br><br>The use of the patient information leaflet reduced the use of antibiotics by nearly a quarter.	Use of antibiotics by patients with acute bronchitis can be reduced with a simple information leaflet.	Leaflet
Wheeler et al.,	Parent attitudes in response to the question 'do you think antibiotics should be used in treating a child with fever and cold?' in those who watched the intervention:<br>- Week 2 – 14.7% stated antibiotics should be used 'always' or 'mostly'.<br>- Week 36 – 9% stated antibiotics should be used 'always' or 'mostly'.<br>In those who did not view the intervention:<br>- Week 2 – 29.1% stated antibiotics	Parent attitudes in response to the question 'did you want/expect the doctor to prescribe antibiotics for your child with a cold and fever?' in those who watched the intervention:<br>- Week 2 – 72.4% stated no.<br>- Week 36 – 86.2% stated no.<br>In those who did not view the intervention:<br>- Week 2 – 63.1% stated no.<br>- Week 36 – 55.7% stated no.	Patients who viewed the videotape intervention were significantly less likely to seek antibiotics for viral infections. However, there was no significant change in antibiotic prescribing by physicians seen despite these changes in patient attitude and expectations. Therefore, the authors conclude that changes in patient attitudes do not seem sufficient for changes in	Video

	should be used 'always' or 'mostly'.<br>- Week 36 – 29.2% stated antibiotics should be used 'always' or 'mostly'.	This study found no change in prescribing habits among physicians during the intervention period, and stated that antibiotic use for viral and bacterial infections remained constant.	antimicrobial prescribing practices.	
Bauchner et al., 2001	A survey of 11 knowledge-based questions had a pre-test average score of 7.50, and a post-test average score of 7.90.<br><br>When comparing the intervention and control groups, unadjusted analyses revealed no significant differences in post-test mean knowledge scores, beliefs or self-reported behaviours. After multivariate analyses and controlling for covariates, there was limited difference seen between the two groups except in 1 behaviour 'I throw out any leftover antibiotic medication' where the intervention group had a significantly greater mean score than the control group (3.82 versus 3.62).<br><br>Post-test mean knowledge scores were significantly higher for the intervention group than for the control group when comparing only participants from the urban clinic location (6.92 versus 6.03), and there was little difference in scores between the groups in the suburban clinic.	The adjusted post-test mean score for the statement 'I ask my paediatrician for an antibiotic even if he/she does not think my child needs one' for the intervention group was 1.15 and 1.25 for the control group.<br><br>No significant differences were found in patient use or expectation of antibiotics between control and intervention groups.	The authors concluded that the impact of the video intervention was modest, and only one difference was found between the intervention and control groups relating to disposing of leftover antibiotics. However, the subgroup analysis demonstrated some more significant effect on participants in the urban clinic groups.	Video
